# Views of Patients on Using mHealth to Monitor and Prevent Diabetic Foot Ulcers: Qualitative Study

**DOI:** 10.2196/diabetes.8505

**Published:** 2017-09-15

**Authors:** Chris Boodoo, Julie Ann Perry, Paul John Hunter, Dragos Ioan Duta, Samuel Carl Paul Newhook, General Leung, Karen Cross

**Affiliations:** 1 Department of Medical Imaging St Michael’s Hospital Toronto, ON Canada; 2 Keenan Research Centre for Biomedical Science St Michael’s Hospital Toronto, ON Canada; 3 Department of Medical Imaging University of Toronto Toronto, ON Canada; 4 Institute of Biomedical Engineering Science & Technology Ryerson University Toronto, ON Canada; 5 Department of Surgery University of Toronto Toronto, ON Canada

**Keywords:** mHealth, telemedicine, diabetic foot ulcer, diabetes, qualitative research

## Abstract

**Background:**

People with diabetes are at risk for diabetic foot ulcers (DFUs), which can lead to limb loss and a significant decrease in quality of life. Evidence suggests that mHealth can be an effective tool in diabetes self-management. mHealth presents an opportunity for the prevention and monitoring of DFUs. However, there is a paucity of research that explores its effectiveness in the DFU patient population, as well as the views and attitudes of these patients toward technology and mHealth.

**Objective:**

This study aimed to explore the views, attitudes, and experiences of a diabetic patient population with or at risk of DFUs regarding technology, mHealth, and the diabetic foot.

**Methods:**

We used a qualitative research approach using in-depth interviews with 8 patients with DFUs. Questions were structured around experience with technology, current health practices related to diabetic foot care, and thoughts on using an mHealth device that prevents and monitors DFUs. We transcribed and thematically analyzed all interviews.

**Results:**

All patients had positive responses for an mHealth intervention aimed at preventing and monitoring DFUs. We found 4 themes in the data: diversity in use of technology, feet-checking habits, 2-way communication with health care professionals (HCPs), and functionality. There were varying levels of familiarity with and dependence on technology within this patient population. These relationships correlated with distinct generations found in North America, including baby boomers and Generation X. Furthermore, we found that most patients performed daily feet checks to monitor any changes in health. However, some did not perform feet checks prior to the development of a DFU. Patients expressed interest in 2-way communication with HCPs that would allow for easier appointment scheduling, sharing of medical data, decreased number of visits, and use of alerts for when medical attention is required. Patients also identified conditions of functionality for the mHealth intervention. These included consideration of debilitating complications because of diabetes, such as retinopathy and decreased mobility; ease of use of the intervention; and implementation of virtual communities to support continued use of the intervention.

**Conclusions:**

Our patient population expressed an interest in mHealth for preventing and monitoring DFUs, although some participants were not frequent users of technology. mHealth continues to show potential in improving patient outcomes, and this study provides a foundation for designing interventions specific to a DFU population. Further research is needed to confirm these findings.

## Introduction

Diabetes affects 1 in 10 people worldwide, many of whom do not have regular access to health care. One of the most devastating consequence of diabetes is the loss of a limb (lower extremity amputation) due to complications resulting from a diabetic foot ulcer (DFU) [1,2]. Diabetic patients have a lifetime risk of 15% to 25% of developing a DFU [3], which can lead to significant decreases in the quality of life, limitations in mobility, function, and independence, and increases in susceptibility to depression and anxiety [2,4]. Moreover, DFUs and lower extremity amputation can lead to loss of livelihood; in 2011, 50% of Canadians with a diagnosis of diabetes were of working age, between 25 and 64 years [5], costing the health care system Can $570 million annually [6]. The prevention of DFUs is of high concern, as the percentage of Canadians at risk for developing diabetes is projected to increase.

The use of mobile technologies in health care is becoming increasingly commonplace. In the United States in 2016, US $867 million was raised by mHealth companies from investors to develop technologies in wearables and sensors, telemedicine, and digital medical devices [7]. mHealth aims to improve the health care service delivery process through support and services to health care providers, or to improve communication between patients and health care services [8]. This makes mHealth a key driver in making health care more accessible to the general population. Over 1100 diabetes-related mHealth apps are available for download, including diabetes self-management and education apps [9]. There is a growing body of evidence supporting the potential for mHealth to have a positive effect on the diabetic population [10-13]. However, there is insufficient evidence on the effectiveness of mHealth interventions for the diabetic foot [14,15]. Also, there is an increase of innovative technology to diagnose DFUs that could be complemented with mHealth, such as infrared thermography and diagnostic algorithms [16,17].

The goal of this study was to explore the views of individuals with DFUs on technology, mHealth, and the diabetic foot.

## Methods

### Design and Ethics

We used a qualitative, descriptive research methodology with face-to-face semistructured interviews to gain in-depth data from consenting patients with diabetes at an outpatient clinic at a tertiary care hospital [18]. We obtained ethics approval from the local institutional research ethics board.

### Study Participants

The study recruited men and women with diabetes from the wound care clinic at a large, tertiary care center. We used a maximum variation purposive sampling approach in this study to maximize diversity and capture common themes relating to the intervention across a range of participants with differing characteristics [18]. Individuals with diabetes received face-to-face invitations to participate in interviews.

### Data Collection

Face-to-face interviews with diabetic patients were conducted by a researcher using a standardized interview guide (Multimedia Appendix 1). A visual pamphlet (Multimedia Appendix 2) and a physical prototype of a DFU prevention and monitoring device developed by an industrial partner was presented during the interview to guide discussion around the development of an mHealth tool. The device is an imaging tool that uses near-infrared light that is attachable to mobile phones. The intended use for the device includes the monitoring of active DFUs and the prevention of DFUs. The only interaction with the device that participants had was during the interview. We conducted interviews rather than focus groups to encourage participants to express opinions that were not influenced by other individuals with diabetes. The content of the interview questions was based on the literature on the evaluation of mobile apps for people with diabetes [19], views of mHealth in a diverse diabetic population [20,21], and the theory of technology adoption [22]. We audiorecorded and transcribed the interviews with each participant’s consent.

### Data Analysis

We used data organization, coding, and thematic analysis to find relationships from the data we collected [18]. We then used an inductive coding approach to develop themes from the raw data [16]. Coding categories were developed and thematic analysis was conducted, using the qualitative analysis software Dedoose (v7.6.6; SocioCultural Research Consultants, LLC), by 2 independent researchers and presented to 2 researchers at our center. Any discrepancies were discussed and resolved to avoid bias in the data.

## Results

### Participants

We invited 7 men and 2 women to participate in the interviews. One man declined participation due to lack of interest in the study. The age of participants ranged from 36 to 77 years with a mean of 53.5 years: 2 participants were older than 65 years, 3 participants were between the ages of 45 and 64 years, and 3 participants were under the age of 44 years. Among the participants, 1 did not have an active DFU, 4 had type 1 diabetes, and 3 did not own a mobile phone, while all participants owned a computer.

### Emergent Themes

All participants were receptive to the concept of an mHealth-based intervention in the prevention and monitoring of DFUs. The participants expressed their views on personal challenges with diabetes and DFUs and their relationship with technology. A total of 4 key themes emerged from the data: diversity in use of technology, feet-checking habits, 2-way communication with health care professionals (HCPs), and functionality.

#### Diversity in Use of Technology

Diabetes and its complications affect individuals of all ages. This makes developing an effective mHealth intervention more difficult, as it needs to target a diverse population. All of our study participants were open to the concept of an mHealth-based intervention in the prevention and monitoring of DFUs. While each participant had a unique relationship with technology, we found correlations between peers of similar age groups and their views on technology.

Participants aged 65 years and older acknowledged that they had limited use for technology. Technology did play a role in their lives, but on a very limited scale. The most frequent use of technology was for communication, such as accessing emails and connecting with friends and family. Both participants described that they did not have the need or use for technology. It did not fit their lifestyles.

I actually don’t do a lot with my phone other than I use it for emails and for phone calls. I am not a techy guy to use my iPhone all the time...It’s a different generation I’m in...I have no need for it. That’s the whole point of technology, it’s gotta suit your needs. And it doesn’t. I don’t need it, so I don’t use it.

Male, 68 years old

Participants younger than 40 years used technology more consistently and frequently. All younger participants owned both a mobile phone and a computer. They described technology as an important source of information and a means to satisfy daily needs, such as generating income, providing entertainment, and connecting with others via social media. This age group expressed the most dependence on technology when compared with other groups in this study.

Middle-aged participants, between the ages of 45 and 65 years, had a less distinctive pattern of technology use and had the most diverse relationship with technology within their own age group. Their experience with technology varied from not owning a mobile phone to frequent daily use of one. Their use of and familiarity with technology related to their daily lifestyle and specific needs. One participant described that he did not own a mobile phone because he spent most of his day in front of his computer and valued the time he had away from it. Another participant had a greater dependence on his mobile phone than did other participants in his age group, but it was not as integrated into his life as it was among participants in the younger age group. He used it as a productivity tool for work, communication, and a few entertainment purposes.

#### Feet-Checking Habits

At-risk people with diabetes are encouraged to check their feet every day for pre-DFU signs and to monitor active DFUs. All participants had a routine they performed for feet checking. Of the 7 participants with active DFUs, 6 checked their feet every day. Most participants inspected their feet themselves, and a few had someone else look at their feet if they could not see, especially the bottom of their feet. Some used a mirror to assist in viewing the bottom of their feet. Others used their hands to feel for abnormalities, and 2 participants mentioned that they checked their feet every day due to the formation of their DFU. This implies that feet checking was not a regular practice prior to the DFU. The participant who did not check his feet every day understood the importance of feet checking but let other priorities prevent him from doing so, such as the responsibilities of being a full-time student.

Although most participants checked their feet, not all had the physical ability to conduct a thorough assessment. The complications from diabetes, such a loss of mobility and poor vision, can prevent individuals from doing a full feet check, especially in the older population. One participant described that some days his joints and muscles were stiffer, which made it more difficult to bend his leg to check his feet. Instead, he would ask his wife to check his feet.

#### Two-Way Communication With Health Care Professionals

mHealth has the potential to enable patients to engage in 2-way communication with their physicians. Most participants were receptive to the idea of being able to communicate with and send physiological readings to their HCP via a mobile phone app. Specifically, they liked the idea of being able to send images of their feet or DFU and receiving an alert of its status. Participants were interested in the convenience of receiving health services from the comfort of their own home and in avoiding going to a clinic if services can be done through mHealth. One participant mentioned the inconveniences of going to the clinic, such as travelling and traffic. Participants also said that they hoped it can assist in contacting their HCP, as some participants had experienced difficulties in doing so in the past. However, a few participants mentioned that the effectiveness of the intervention would depend on the physician’s response time on the app. If a timely response cannot be guaranteed, then the participants would not want to use the app. They described some negative consequences, such as untimely care for health issues and introducing unnecessary worry to patients stemming from the wait for a response.

Although interest was high among participants, older individuals also expressed that they would not want their doctors to be replaced by mHealth. They preferred in-person visits and mentioned that it’s what their generation is used to. They also valued the relationship between the doctor and the patient, and described it as an integral part of care given to patients. One participant described the value of in-person interactions with his doctor.

No, I would still want to go to [doctor name] every 3 weeks, or whatever he feels appropriate. He’s a great guy, we’re very good friends...the relation between the patient and the doctor is—well I’ve been very spoiled, but I know what it can be—it’s crucial.

Male, 77 years old

#### Functionality

For mHealth interventions to be successful, certain factors about the targeted population must be considered to increase adoption. Participants provided feedback on the presented prototype and mentioned specific features that can increase the usability of the device. One of the most frequent suggestions was to maximize ease of use, as this device might also be used by older patients. A few participants suggested being trained by a professional before being given the device, or to have a manual provided with the device to assist in learning to use the novel technology. Two participants made recommendations for such interventions to better suit the needs of individuals living with complications from diabetes, as they can impair use of a device designed for someone without diabetic complications. One participant suggested the use of bright colors in the app to overcome vision issues caused by retinopathy. Another suggestion was to consider the decreased mobility in individuals with diabetes, which could limit people’s use of this mHealth technology if it depended on certain functional movements, such as taking an image of the user’s foot.

Encouraging prolonged use of an mHealth intervention is another challenge that must be considered in a DFU population. Some participants expressed ideas on how to maintain interest in and use of the device, as depression has been linked to people with diabetes. Some participants suggested incorporating some sort of community into the app, as this can keep users excited to continue self-reporting and to stay updated with other users’ status. This would provide another reason to keep using the device that is not just centered on the patient’s health.

## Discussion

This qualitative study explored attitudes and perceptions toward technology and mHealth in individuals with and at risk of DFUs and reflected on specific conditions from the patient’s perspective for increased mHealth adoption in diabetic foot care. We found that individuals responded positively to an mHealth intervention aimed at preventing and monitoring their DFUs. This study contributes to the literature by identifying potential users’ DFU-related practices and by highlighting issues related to the development and integration of mHealth interventions.

Preventing and treating DFUs requires patients with diabetes to be proactive in the care of their own feet, including carefully checking their feet daily for preulcerative signs and wound healing [23]. Current efforts, such as multidisciplinary clinics and patient education, are found to be cost effective, as they relieve the economic burden on the health care system by preventing DFUs and providing higher standards of care [24-26]. However, there is no systematic foot screening program in place for at-risk diabetic patients. Therefore, innovative solutions are required to improve the systems in place for DFU prevention and care, and ideally to focus on mechanisms that would allow for thorough foot assessments outside of the clinical setting. Leveraging the strengths of mHealth may improve current patient practices in DFU prevention and care; although the evidence is mixed, there appears to be potential to increase adherence to chronic disease management [27]. In addition, caution should be taken when implementing similar interventions in this population, as individuals with DFUs are at a much higher risk of morbidity and death [28]. Future studies should further explore the effectiveness of the use mHealth for DFU prevention and care using larger sample sizes and longer follow-ups.

This study focused on participants’ current DFU monitoring practices. All participants were conscious about their foot health, and most patients checked their feet every day. We found that the thoroughness of a participant’s foot checks depended on their mobility and stage of diabetes. If an individual had poor mobility, increased joint stiffness, or vision problems, it was more difficult for them to inspect all parts of their feet. Some required aids such as mirrors or having someone else inspect their feet. Moreover, some did not start checking their feet daily until the formation of a DFU, which may reflect a lack of education regarding the need for monitoring rather than treatment when it comes to the development of DFUs with diabetes. Future studies should explore the daily feet-checking routine of at-risk people with diabetes to further explore potential barriers in this crucial practice.

We also identified varying levels of experience with technology among our study participants. This is consistent with the fact that DFUs affect individuals with diabetes of all ages [29]. Varying levels of familiarity with technology in a target population poses a challenge in satisfying the needs of the target users [30,31]. Specifically, older participants displayed lower familiarity with technology. This may be due to a digital divide, where older people tend to be excluded from benefiting from Internet technology [32]. This lack of familiarity with technology has been identified by other studies as a barrier to mHealth adoption [30,31,33,34], and suggests that future studies should explore how mHealth can be effective in a population where familiarity with technology is diverse.

We also identified conditions for a successful mHealth tool from the perspective of an individual with a DFU. Participants identified ease of use as an important factor, which has also been mentioned in other studies where rates of adoption increased among baby boomers and older generations [30,33,35,36]. Ease of use encourages adoption among individuals who are not familiar with technology and prevents early negative experiences that may discourage the use of the intervention [22]. Mobility and vision problems were mentioned frequently in our study (and are frequent in people with diabetes), and technology designed for this patient population must take these challenges into account [33,37]. Depression was also mentioned as a factor that can prevent mHealth adoption, and is associated with lower health-related quality of life and higher mortality in a diabetic population [38,39]. Participants suggested that communities and online forums should be integrated into mHealth interventions to encourage initial and continued use. This would allow patients to share their thoughts and experiences and to develop relationships for peer support. Health-related virtual communities have been found to be an effective way to provide information to members and provide socioemotional support where members gained psychosocial benefits, although more evidence is required to confirm this [40]. However, this does not come without the inherited risks of social media. Risks include poor quality of information shared between patients, which can lead to deviation from a professional’s advice in care and risks of patient privacy breaches [41]. Therefore, careful considerations must be made when implementing such communities by including measures to protect both patients and HCPs [41,42].

Participants in this study also expressed interest in a 2-way communication channel with an HCP via the mHealth intervention. They expressed that having this channel would allow for easier appointment scheduling, sharing of medical data, decreased number of visits, and use of alerts for required medical attention. Providing a way for patients to communicate with their HCP would make access to health care services more pervasive, especially for people living in remote areas or people with decreased mobility. A review of telemedicine technologies that require an HCP to respond, either in real time or with a delay, to the clinical information transmitted via telemedicine found that it has the potential to be an effective tool for delivering more frequent and timely health care to people with chronic conditions at a distance and for improving access to health care [43]. This shows that 2-way communication with an HCP can improve health outcomes of individuals with DFUs. However, some older participants were reluctant to have technology replacing their HCPs. Future research should explore how to best implement this to ensure effective communication between patients and HCPs while sustaining the patient-doctor relationship.

Although the considerations mentioned above have been found to be important in the development of an mHealth tool, how it is implemented into an HCP’s workflow is crucial for its success [44]. The adoption of such interventions does not come without change in the routine of HCPs and must be considered. Two studies qualitatively investigated conditions that would need to be addressed for successfully introducing telemedicine in diabetes foot care from the perspective of HCPs [45,46]. These included training for HCPs, concerns for whether an mHealth approach would reduce the hands-on skills and multidisciplinary approach specialized in wound care, change to communication channels within the clinical environment, and having a telemedicine champion in the work setting [45,46]. Consequently, the development of mHealth tools must consider the impact on both patients and HCPs.

### Strength and Limitations

A strength of this study was the approach to include patients of all ages with DFUs, which provided the study with an accurate representation of the perspective of a typical DFU population. Conversely, having a wide variation of participants can be a limitation, as it weakens the content saturation of the study. Future research should increase the sample size or recruit individuals from similar age groups to increase content saturation in this field.

A limitation of this study was also its relatively small sample size. This may decrease the strength of the results presented. However, the exploratory objective of this study will hopefully influence future studies with larger sample sizes in this field.

Participants were also exposed to the mHealth device only during the interview session. Therefore, their views were based solely on this interaction and information from the interviewer via the pamphlet and conversation. However, the results presented are focused on their views on mHealth in general rather than direct feedback on the prototype itself.

We recruited participants from only 1 wound care clinic, and our findings cannot be generalized outside the population in this sample due to regional differences. However, regions with similar characteristics may offset these limitations. The small sample size further limits the generalizability of our findings. This study did not capture the views of individuals who are not active in the health system, and our findings may not represent their views. The findings from this study are accounts of the views of the sample’s perspective on mHealth, technology, and the diabetic foot.

### Conclusions

The population we studied expressed generally positive views on mHealth for preventing and monitoring DFUs. This indicates the potential for mHealth to improve health outcomes for individuals with and at risk of DFUs. Although only a small portion of the patient population were using technology for health reasons, they were open to the idea of using an mHealth technology if it would improve their quality of life. This is an important indicator that mHealth may be a platform for solutions moving forward as the health care system continues to be burdened by patients with diabetic complications. This study further improves on the understanding of opportunities and challenges of developing an mHealth intervention for individuals with diabetes and provides a foundation for interventions specific to a DFU population.

## Appendix

Multimedia Appendix 1 Interview guide used by the researcher during the one-on-one interviews with participants.

Multimedia Appendix 2 Pamphlet shown to participants by the researcher to assist in the explanation of the presented mHealth prototype device.

## Abbreviations

DFU: diabetic foot ulcer
HCP: health care professional
