# Supplementary material for: Views of Patients on Using mHealth to Monitor and Prevent Diabetic Foot Ulcers: Qualitative Study
Source: JMIR Diabetes. 2017 Sep 15;2(2):e22. doi: 10.2196/diabetes.8505 (PMC6238864; doi:10.2196/diabetes.8505)
Supplement: Multimedia Appendix 1 [file diabetes_v2i2e22_app1.pdf]

Study title: Assessment of Wound Viability in the Lower Extremity of Diabetics by Multi-Spectral Imaging and MRI

Patient ID #_________

STUDY QUESTIONNAIRE

1. How often do you usually check your feet?

☐ Every day ☐ Once a week ☐ Never ☐ Other (please specify) _____________

Probe:

- What types of challenges do you encounter, if any, during this process?

2. On a scale of 1 to 10, how easy was [DEVICE] to use **in general**? (please circle)

Very easy 1 2 3 4 5 6 7 8 9 10 Hard

3. If [DEVICE] was hard to use **in general**, what would make it easier?

Probes:

- Can you describe any difficulty that you had or may have in imaging your foot with this device?

4. On a scale of 1 to 10, how easy was the [DEVICE] '**app**' to use? (please circle)

Very easy 1 2 3 4 5 6 7 8 9 10 Hard

5. If the [DEVICE] '**app**' was hard to use, what would make it easier?

Probes:

- Maybe having the camera and phone separated, so imaging the bottom of your foot is easier?

6. Do you think using [DEVICE] will save you from having to go to the wound care clinic so often?

☐ Yes ☐ No ☐ No change

7. Now, I want to talk about your relationship with technology. Do you own a smartphone or a tablet? If so, which one?
    - How did you obtain your phone?
    - If not, how about a computer?

8. What do you use your phone for?
    - What applications do you use?
    - Are there any apps you use daily?
    - Any apps that you use for health reasons?
    - How do you decide on using an app, and continue using it?
    - Is your phone a source of information for you?

9. Does your smartphone have a camera?
    - Do you use it often?
    - Describe your experience with the camera interface.
    - Do you have issues locating images on your phone after taking them?

10. Thank you for completing the survey. Please let us know if you have any further comments about [DEVICE] in the space below.
