# Supplementary material for: Views of Patients on Using mHealth to Monitor and Prevent Diabetic Foot Ulcers: Qualitative Study
Source: JMIR Diabetes. 2017 Sep 15;2(2):e22. doi: 10.2196/diabetes.8505 (PMC6238864; doi:10.2196/diabetes.8505)
Supplement: Multimedia Appendix 2 [file diabetes_v2i2e22_app2.pdf]

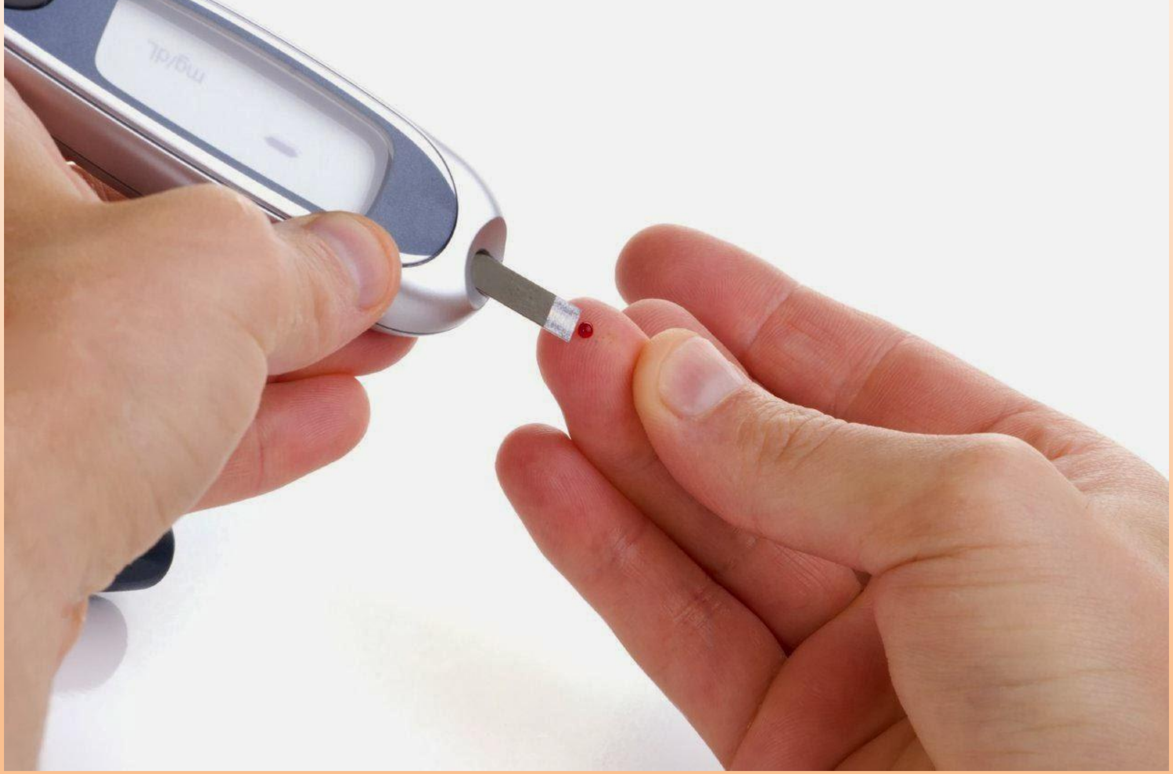

Your blood sugar is **monitored** using a glucometer

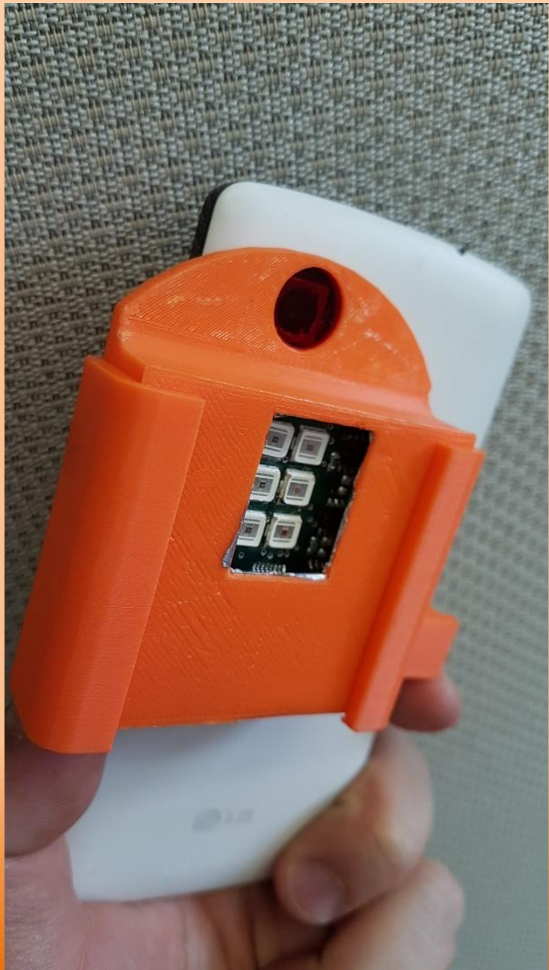

We are designing a device to monitor your **foot health**.

Using the [DEVICE] is just like taking a picture with your cell phone. It should be used as a part of your diabetes monitoring routine, like your glucometer.

Feature	Glucometer	[DEVICE]
Used daily	✓	✓
Simple	✓	✓
Painless	✗	✓
Collects health data	✓	✓
Helps control complications of your diabetes	✓	✓
Connects you to your doctor remotely	✗	✓
Does not replace your doctor	✓	✓

We are trying to design the [DEVICE] to be as easy to use as possible, and we'd like your help!
